# Hepatic overexpression of protein targeting to glycogen attenuates obesity and improves hyperglycemia in db/db mice

**DOI:** 10.3389/fendo.2022.969924

**Published:** 2022-09-09

**Authors:** Iliana López-Soldado, Joan J. Guinovart, Jordi Duran

**Affiliations:** ^1^ Institute for Research in Biomedicine (IRB Barcelona), The Barcelona Institute of Science and Technology, Barcelona, Spain; ^2^ Centro de Investigación Biomédica en Red de Diabetes y Enfermedades Metabólicas Asociadas (CIBERDEM), Madrid, Spain; ^3^ Department of Biochemistry and Molecular Biomedicine, University of Barcelona, Barcelona, Spain; ^4^ Institut Químic de Sarrià (IQS), Universitat Ramon Llull (URL), Barcelona, Spain; ^5^ Institute for Bioengineering of Catalonia (IBEC), The Barcelona Institute of Science and Technology, Barcelona, Spain

**Keywords:** glycogen, glucose, liver, food intake, db/db, ATP

## Abstract

Increased liver glycogen content has been shown to reduce food intake, attenuate obesity, and improve glucose tolerance in a mouse model of high-fat diet (HFD)-induced obesity. Here we studied the contribution of liver glycogen to the regulation of obesity and glucose metabolism in a model of type 2 diabetes and obesity, namely the db/db mouse. To this end, we crossed db/db mice with animals overexpressing protein targeting to glycogen (PTG) in the liver to generate db/db mice with increased liver glycogen content (db/db-PTG). Hepatic PTG overexpression reduced food intake and fat weight and attenuated obesity and hyperglycemia in db/db mice. Db/db-PTG mice showed similar energy expenditure and physical activity to db/db mice. PTG overexpression reduced liver phosphoenolpyruvate carboxykinase (PEPCK) protein levels and repressed hepatic glucose production in db/db mice. Moreover, increased liver glycogen elevated hepatic ATP content in these animals. However, lipid metabolism was not modified by PTG overexpression. In conclusion, increased liver glycogen content ameliorates the diabetic and obesity phenotype in db/db mice.

## Introduction

The emergence of type 2 diabetes mellitus (T2DM) as a global pandemic poses a major challenge for human health in the 21^st^ century (1). To gain an appreciation of the numbers involved, in 2017, approximately 462 million individuals were affected by T2DM (2).

The liver governs various pathways of glucose metabolism and therefore plays a major role in the regulation of glucose homeostasis (3). These hepatic processes are dysregulated in T2DM, and this imbalance contributes to hyperglycemia in the fasted and postprandial states (4). Liver glycogen metabolism is pivotal in glucose homeostasis. In this regard, postprandial liver glycogen synthesis is reduced in mildly overweight T2DM patients (5). Glycogen synthesis is regulated mainly by glycogen synthase, which is dephosphorylated and thus activated by protein phosphatase 1 (PP1) in combination with glycogen-targeting subunits or G subunits, which localize PP1 to glycogen particles. Seven G subunits, among these proteins targeting to glycogen (PTG, also known as PPP1R3C or PPP1R5) (6), regulate glycogenesis in different organs. PTG overexpression in a variety of cell types and in rodent livers *in vivo* results in a dramatic increase in cellular glycogen accumulation (6–8).

We previously demonstrated that, when fed a high-fat diet (HFD), mice that overexpress PTG in the liver show a reduced food intake compared to control mice. This resulted in lower body weight, decreased fat mass, and reduced leptin levels (8). Furthermore, PTG overexpression reversed the glucose intolerance caused by the HFD (8). However, it is important to understand whether the positive effect of increasing liver glycogen was limited to the HFD model or whether it could be extended to other models of T2DM. To address this question, here we studied the effect of increasing liver glycogen content in db/db mice, another widely used model of T2DM and obesity. Various mechanisms underlie T2DM in HFD-induced obese mice and db/db mice. The latter carry a spontaneous mutation in the leptin receptor (9) and suffer hyperphagia, severe glucose intolerance, hyperglycemia, hyperleptinemia and hyperinsulinemia. In contrast, the former have milder glucose intolerance and other metabolic symptoms, and this phenotype is reversible with a low-fat diet (10). Our results show that increased liver glycogen stores attenuate obesity and improves hyperglycemia also in the context of db/db mice.

## Materials and methods

### Experimental animals

All procedures were approved by the Barcelona Science Park’s Animal Experimentation Committee and were carried out following the European Community Council Directive and the National Institute of Health guidelines for the care and use of laboratory animals. Db/db mice (strain name BKS.Cg-*Dock7^m^* +/+ *Lepr^db^*/J) were purchased from the Jackson Lab. The db/db mice were backcrossed to C57BL/6J for 8 generations and then mated with Cre-Albumin mice on the C57BL/6J background that overexpressed hepatic PTG. Mice overexpressing PTG specifically in the liver were generated as previously described (8, 11). Heterozygous db/+ mice were used as controls. Since the PTG expression cassette was introduced into the Hprt locus in the X chromosome (8), and to avoid variability due to female X chromosome inactivation, all the studies were conducted in male animals. All the mice studied were littermates. Animals were sacrificed at 18 weeks of age under ad libitum-fed conditions between 8 and 10 a.m. by cervical dislocation, and tissues were collected and frozen in liquid nitrogen. Whole blood was collected from tails in EDTA-coated tubes and then centrifuged, and plasma was stored at –20°C for analysis.

### Body mass composition

Lean weight and fat weight were measured by magnetic resonance imaging (EchoMRI System, EchoMRI LLC, Houston, TX, USA).

### Metabolic activity

Indirect calorimetry was performed using an eight-chamber Oxymax system (Columbus Instruments) to measure energy expenditure, which was calculated from oxygen consumption and CO_2_ production. Mice were allowed to acclimatize to the cages for 2 days before three cycles of 24-h measurements. Energy expenditure (standardized for body weight) was calculated as EE = (3.185 + 1.232 × RER) × *V*_O2_, respiratory exchange ratio (RER) was calculated as *V*_CO2_/*V*_O2_, glucose oxidation was calculated in g/min/kg^0.75^ = [(4.545 × *V*co_2_) − (3.205 × *V*o_2_)]/1000 and lipid oxidation was calculated in g/min/kg^0.75^ = [1.672 × (*V*o_2_ − *V*co_2_)]/1000). Ambulant and total locomotor activity was monitored by an infrared photocell beam interruption method.

### Food consumption

To monitor food intake, mice were housed individually and acclimatized for a week before the study. Food intake was measured daily for 5 consecutive days.

### Blood and biochemical analysis

For liver glycogen quantification, frozen tissue was homogenized in 4 volumes of 30% KOH at 4°C. The homogenate was heated at 100°C for 15 min. A volume of 50 µl of the heated homogenate was deposited in 31ET paper (Whatman, Maidstone, UK). Glycogen precipitates in the 31ET paper due to immersion in cold ethanol 66% (v/v) for 10 min. After two washes with 66% ethanol for 20 min at room temperature, papers were washed with acetone and left air drying overnight. Dry papers were incubated for 90 min at 37°C with 25 mg/ml of amyloglucosidase (A7420, Sigma-Aldrich, St Louis, USA) diluted in 100 mM sodium acetate buffer at pH 4.8. After the incubation, the resulting solution was collected into new tubes and used to measure glucose concentration using a commercial kit (Horiba, ABX, Montpellier, France). Hepatic nucleotides (ATP and AMP) were measured by HPLC in perchloric acid extracts. Briefly 75 mg of frozen tissue was homogenized in 0.75 ml of 10% HClO_4_. Samples were spun at 3,500 *g* at 4°C for 20 min. The supernatant was neutralized with 0.5 M K_2_CO_3_. Samples were placed on ice for 15 min and spun at 3,500 *g* at 4°C for 15 min. Analysis was performed using a Brisa LC2 C18 column (4.6 × 150 mm, 3-μm particle size) interfaced with a Photodiode Array Detector (PDA) detector (260 nm) and a constant flow rate of 0.6 ml/min. ATP was identified using a gradient of mobile phase (70:30 ratio of eluent A/MeOH) for 37 min and quantified using known standards [Eluent A (500 mM monopotassium phosphate (KH_2_PO_4_) + 4 mM tetramethylammonium hydrogen sulfate (C_4_H_12_HSO_4_ pH=6)]. Hepatic triacylglycerol (TAG) was quantified in 3 mol/L KOH and 65% ethanol extracts following the method described by Salmon and Flatt (12) and using a kit (Sigma-Aldrich, St Louis, MO, USA). Plasma insulin was measured by ELISA using a commercial kit (Crystal Chem, Elk Grove Village, IL, USA). Blood glucose was determined using a glucometer (Bayer Contour Next, Bayer Healthcare, Leverkusen, Germany).

### Western blot (WB) analysis

Liver samples were homogenized in 50 mM Tris/HCl (pH 7.4), 150 mM NaCl, 1 mM EDTA, 5 mM sodium pyrophosphate, 1 mM sodium orthovanadate, 50 mM NaF, 1% NP-40, 1 mM PMSF, and a protease inhibitor cocktail tablet (Roche, Basel, Switzerland). Homogenized tissues were incubated on ice for 30 min. Samples were then centrifuged for 15 min at 12,000 rpm at 4°C and supernatants were transferred to clean vials. Protein concentration was measured using the bicinchonic acid (BCA) protein assay reagent (Thermo Fisher Scientific, Massachusetts, USA). Samples were loaded on 10% acrylamide gels for SDS-PAGE and transferred to Immobilon membranes (Millipore, Sigma-Aldrich, St Louis, MO, USA). Immunoblot analysis of homogenates was performed using the following antibody: PEPCK (a kind gift from Dr. E. Beale) dilution 1:100.000. Proteins were detected by the ECL method (Immobilon Western Chemoluminescent HRP Substrate, Millipore, Sigma-Aldrich, St Louis, MO, USA). The loading control of the WB membrane was performed using the REVERT total protein stain.

### Glucose tolerance test

The glucose tolerance test (GTT) was performed in 16-h fasted mice after an intraperitoneal injection of 1 g/kg of body weight glucose bolus. Blood glucose was measured at the indicated time points after the challenge.

### Insulin tolerance test

The insulin tolerance test (ITT) was performed in 6-h fasted mice after an intraperitoneal injection of 0.75 units/kg for db/+ and db/+-PTG or 2 units/kg for db/db and db/db-PTG. Blood glucose was measured at the indicated time points after the challenge.

### Pyruvate tolerance test

The pyruvate tolerance test (PTT) was performed in 16-h fasted mice after an intraperitoneal injection of 2 g/kg of body weight of sodium pyruvate (Sigma-Aldrich, St Louis, MO, USA). Blood glucose was measured at the indicated time points after the challenge.

### RNA extraction and quantitative RT-PCR

For liver RNA extraction, 50 mg of the frozen sample was homogenized in 500 µl of TRIzol (Invitrogen, Waltham, MA, USA). After 5 min of incubation at room temperature, 0.1 ml of chloroform was added. Samples were centrifugated at 12,000 rpm for 15 min at 4°C. The upper phase was collected into a new tube and 0.3 ml of 70% ethanol was added. The mix was transferred into columns from the RNeasy Micro Kit (Qiagen, Hilden, Germany) and RNA was isolated following the manufacturer’s instructions. Single-stranded complementary DNA was produced by reverse transcription using 1 µg of RNA in a 20-µL reaction qScript cDNA SuperMix (Quanta bio, Beverly, MA, USA). Quantitative polymerase chain reaction (PCR) was performed using Taqman universal PCR master mix (Applied Biosystem, Waltham, MA, USA) on the QuantStudio 6 Flex (Applied Biosystem, Waltham, MA, USA). The ΔCt was defined as the difference between the Q-PCR cycles of the housekeeping gene and those of the target genes. The following TaqMan probes (Applied Biosystems, Waltham, MA, USA) were used for quantitative real-time PCR: PTG (Mm01204084_m1); ACC1 (Mm01304257_m1); FAS (Mm00662319_m1); SCD1 (Mm00772290_m1); GPAT1 (Mm00833328_m1); and 18S (Mm03928990_g1). 18S was used as a housekeeping gene.

### Statistical analysis

Data are expressed as mean ± SEM. P values and F ratio were calculated using two-way ANOVA with *post hoc* Tukey tests as appropriate using Prism8 software (GraphPad).

## Results

### Hepatic PTG overexpression decreases body weight and food intake in db/db mice

**Figure 1:**
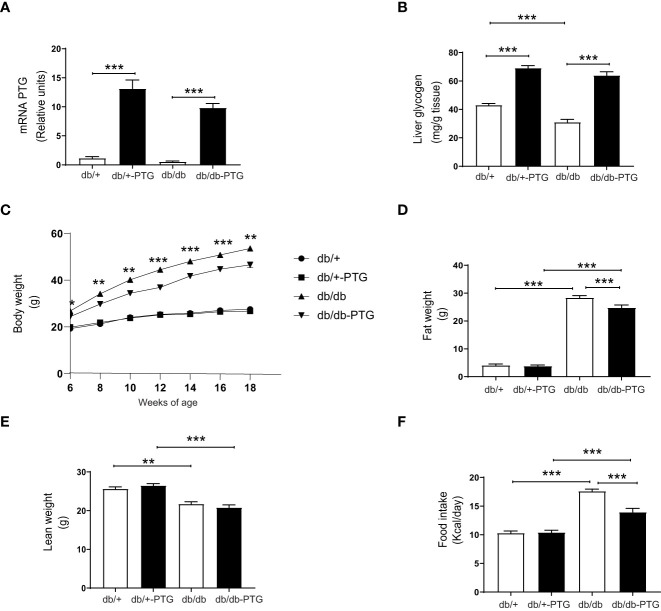
Liver PTG overexpression decreases body weight and food intake in db/db mice. **(A)** Relative mRNA of PTG in the liver, **(B)** Liver glycogen content, **(C)** Growth curve from 6-18 weeks of age, **(D)** Fat weight, **(E)** Lean weight, and **(F)** Daily food intake of db/db and db/db-PTG mice, and non-diabetic controls (n=6-12 in all experiments except food intake n=18-26). All values are mean ± SEM. *P<0.05, **P<0.01, ***P<0.001. For figure **(A)** Diabetes factor (F ratio=4.090, P=0.0540), PTG factor (F ratio=123.5, P<0.0001), Interaction (F ratio=1.963, P=0.1734). For figure **(B)** Diabetes factor (F ratio=20.81, P<0.0001), PTG factor (F ratio=243.8, P<0.0001), Interaction (F ratio=3.354, P=0.0777). For figure **(C)** 6 weeks of age Diabetes factor (F ratio=43.79, P<0.0001), PTG factor (F ratio=2.250, P=0.1456), Interaction (F ratio=5.106, P=0.0325). For figure **(C)** 8 weeks of age Diabetes factor (F ratio=119.5, P<0.0001), PTG factor (F ratio=3.982, P=0.0566), Interaction (F ratio=8.517, P=0.0072). For figure **(C)** 10 weeks of age Diabetes factor (F ratio=175.3, P<0.0001), PTG factor (F ratio=8.286, P=0.0079), Interaction (F ratio=8.461, P=0.0073). For figure **(C)** 12 weeks of age Diabetes factor (F ratio=327.4, P<0.0001), PTG factor (F ratio=19.51, P=0.0002), Interaction (F ratio=20.02, P=0.0001). For figure **(C)** 14 weeks of age Diabetes factor (F ratio=467.6, P<0.0001), PTG factor (F ratio=94.71, P=0.0004), Interaction (F ratio=13.08, P=0.0013). For figure **(C)** 16 weeks of age Diabetes factor (F ratio=529.7, P<0.0001), PTG factor (F ratio=20.50, P=0.0001), Interaction (F ratio=15.65, P=0.0005). For figure **(C)** 18 weeks of age Diabetes factor (F ratio=442.1, P<0.0001), PTG factor (F ratio=9.595, P=0.0046), Interaction (F ratio=7.617, P=0.0105). For figure **(D)** Diabetes factor (F ratio=1620, P<0.0001), PTG factor (F ratio=11.46, P=0.0017), Interaction (F ratio=14.32, P=0.0005). For figure **(E)** Diabetes factor (F ratio=62.29, P<0.0001), PTG factor (F ratio=0.4716, P=0.4964), Interaction (F ratio=6.722, P=0.0134). For figure **(F)** Diabetes factor (F ratio=128.8, P<0.0001), PTG factor (F ratio=13.47, P=0.0004), Interaction (F ratio=15.02, P=0.0002).

PTG was overexpressed specifically in the liver of male db/+ and db/db mice. As expected, the mRNA level of PTG was increased in db/+-PTG and db/db-PTG mice (**Figure 1A**). The liver glycogen concentration in db/db mice was significantly lower than in control mice (db/+) (**Figure 1B**), as previously described (13). As expected, the overexpression of PTG in the liver caused a significant increase in liver glycogen concentration in the db/+-PTG and db/db-PTG groups compared to db/+ and db/db animals, respectively (**Figure 1B**).

Body weights were recorded from 6 weeks of age till the end of the experiment (18 weeks of age). At week 6, the db/db and db/db-PTG animals were heavier than their respective controls. From week 6 to week 18, db/db-PTG mice weighed significantly less than db/db counterparts. There were no differences in body weight between db/+ and db/+-PTG mice throughout the study (**Figure 1C**). At the end of the 16 weeks, we analyzed the total body fat and lean mass for all groups by magnetic resonance imaging. Diabetic mice of both genotypes showed a higher fat mass and lower lean mass than non-diabetic counterparts (**Figures 1D, E**). Db/db-PTG mice had a significantly lower total fat mass than db/db mice (**Figure 1D**). However, the lean mass was similar between the two genotypes (**Figure 1E**).

**Figure 2:**
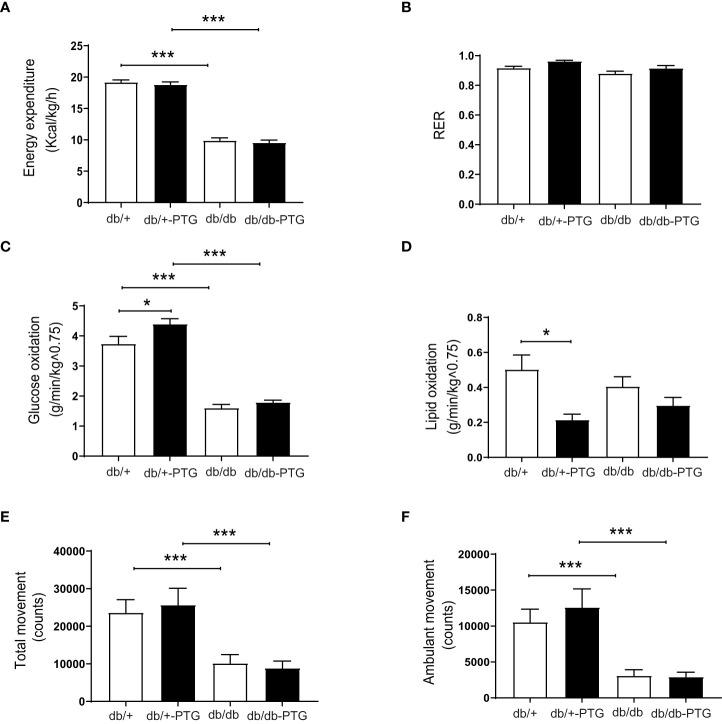
Energy expenditure and physical activity are similar in db/db-PTG and db/db mice. **(A)** Energy expenditure, **(B)** RER, **(C)** Glucose oxidation, **(D)** Lipid oxidation, **(E)** Total movement, and **(F)** Ambulant movement of db/db and db/db-PTG mice, and non-diabetic controls (n=6-9 in all experiments). All values are mean ± SEM. *P<0.05, ***P<0.001. For figure **(A)** Diabetes factor (F ratio=532.9, P<0.0001), PTG factor (F ratio=0.7677, P=0.3887), Interaction (F ratio=0.001914, P=0.9654). For figure **(B)** Diabetes factor (F ratio=8.282, P=0.0077), PTG factor (F ratio=7.750, P=0.0097), Interaction (F ratio=0.1423, P=0.7090). For figure **(C)** Diabetes factor (F ratio=239.8, P<0.0001), PTG factor (F ratio=7.535, P=0.0106), Interaction (F ratio=2.352, P=0.1368). For figure **(D)** Diabetes factor (F ratio=0.02006, P=0.8885), PTG factor (F ratio=12.10, P=0.0019), Interaction (F ratio=2.466, P=0.1289). For figure **(E)** Diabetes factor (F ratio=25.38, P<0.0001), PTG factor (F ratio=0.01504, P=0.9033), Interaction (F ratio=0.3059, P=0.5848). For figure **(F)** Diabetes factor (F ratio=31.68, P<0.0001), PTG factor (F ratio=0.3834, P=0.5410), Interaction (F ratio=0.5392, P=0.4691).

To determine the cause of the lower body weight and body fat in db/db-PTG mice, we examined their daily food intake and energy expenditure. Daily food intake was higher in diabetic compared to non-diabetic mice (**Figure 1F**), but db/db-PTG mice ate less than db/db mice, which may explain the lower body weight of the former (**Figure 1F**). We studied metabolic parameters by means of indirect calorimetry using the Oxymax system. Mice were placed in individual metabolic chambers for 48 h, and gas exchange and activity were monitored. Energy expenditure was lower in diabetic than non-diabetic mice. However, there were no differences in energy expenditure between db/db and db/db-PTG mice (**Figure 2A**). The respiratory exchange ratio (RER), an indicator of the fuel (i.e. carbohydrate or fat) being metabolized to supply the body with energy, was similar between all the groups analyzed (**Figure 2B**). Regarding glucose oxidation, diabetic mice oxidized less glucose than non-diabetic counterparts (**Figure 2C**). Furthermore, the db/+-PTG mice oxidized more glucose and less lipids than the db/+ mice (**Figures 2C, D**). However, no differences were found between the db/db and db/db-PTG groups. Greater physical activity of the db/db-PTG group may also contribute to their lower body weight compared to db/db mice. To explore this notion, total movement and ambulant movement were measured. Diabetic mice moved less than non-diabetic counterparts but no differences between db/db and db/db-PTG mice were observed (**Figures 2E, F**).

### Hepatic PTG overexpression reverts hyperglycemia in db/db mice

**Figure 3:**
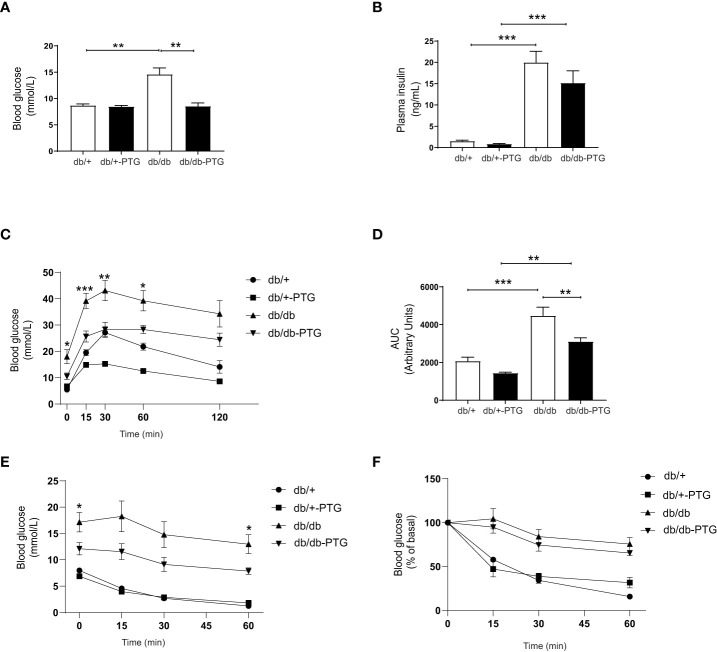
Hepatic overexpression of PTG reverts hyperglycemia in db/db mice. **(A)** Blood glucose, **(B)** Plasma insulin, **(C)** GTT curve for glucose, **(D)** AUC for glucose in the GTT, **(E)** ITT curve for glucose, and **(F)** ITT curve for glucose expressed as the percentage of initial values for db/db and db/db-PTG mice, and non-diabetic controls (n=6-14 in all experiments). All values are mean ± SEM. *P<0.05, **P<0.01, ***P<0.001. For figure **(A)** Diabetes factor (F ratio=7.195, P=0.0118), PTG factor (F ratio=7.150, P=0.0110), Interaction (F ratio=7.002, P=0.0118). For figure **(B)** Diabetes factor (F ratio=57.71, P<0.0001), PTG factor (F ratio=1.594, P=0.2135), Interaction (F ratio=0.9109, P=0.3452). For figure **(C)** time 0, Diabetes factor (F ratio=18.06, P=0.0002), PTG factor (F ratio=2.654, P=0.1149), Interaction (F ratio=5.059, P=0.0329). For figure **(C)** time 15, Diabetes factor (F ratio=40.23, P<0.0001), PTG factor (F ratio=14.31, P=0.0008), Interaction (F ratio=3.444, P=0.0744). For figure **(C)** time 30, Diabetes factor (F ratio=20.27, P=0.0001), PTG factor (F ratio=17.46, P=0.0003), Interaction (F ratio=0.1980, P=0.6599). For figure **(C)** time 60, Diabetes factor (F ratio=34.92, P<0.0001), PTG factor (F ratio=12.84, P=0.0013), Interaction (F ratio=0.08646, P=0.7710). For figure **(C)** time 120, Diabetes factor (F ratio=22.24, P<0.0001), PTG factor (F ratio=4.044, P=0.0544), Interaction (F ratio=0.3171, P=0.5780). For figure **(D)** Diabetes factor (F ratio=34.95, P<0.0001), PTG factor (F ratio=12.59, P=0.0014), Interaction (F ratio=0.5225, P=0.4760). For figure **(E)** time 0, Diabetes factor (F ratio=37.17, P<0.0001), PTG factor (F ratio=6.741, P=0.0153), Interaction (F ratio=2.575, P=0.1090). For figure **(E)** time 15, Diabetes factor (F ratio=36,54, P<0.0001), PTG factor (F ratio=4.318, P=0.0477), Interaction (F ratio=3.019, P=0.0941). For figure **(E)** time 30, Diabetes factor (F ratio=37.52, P<0.0001), PTG factor (F ratio=3.314, P=0.0802), Interaction (F ratio=3.768, P=0.0632). For figure **(E)** time 60, Diabetes factor (F ratio=71.07, P<0.0001), PTG factor (F ratio=4.625, P=0.0410), Interaction (F ratio=7.297, P=0.0120).

The liver plays a key role in the maintenance of glucose homeostasis—it produces glucose during fasting and stores glucose after eating. Blood glucose homeostasis can be improved by modulating the expression or activity of proteins involved in the regulation of liver glycogen metabolism (14). Interestingly, PTG overexpression led to a decrease in glucose concentrations levels in db/db mice (**Figure 3A**). Plasma insulin was similarly increased in db/db mice and db/db-PTG mice (**Figure 3B**). This observation might be attributable to the absence of leptin receptor in these mice, which is associated with severe persistent hyperinsulinemia (15). To further evaluate the effect of hepatic PTG overexpression on glucose metabolism, we performed an intraperitoneal glucose tolerance test (GTT). The plasma glucose concentration during the GTT was markedly decreased at 0, 15, 30 and 60 min after intraperitoneal injection of glucose in db/db-PTG mice compared to db/db animals (**Figure 3C**). The area under the curve (AUC) was significantly smaller in the db/db-PTG group than in the db/db group (**Figure 3D**). These results demonstrated that the former had improved glucose homeostasis. To determine possible changes in insulin sensitivity, an intraperitoneal insulin tolerance test (ITT) was performed. After a 6-h fast, db/db-PTG had significantly lower blood glucose concentration than db/db mice (**Figure 3E**), which made the analysis of the results difficult to interpret. Therefore, the ITT was expressed as the percentage of the initial values, and no differences were observed among db/db and db/db-PTG (**Figure 3F**), indicating that insulin resistance was similar in both groups of diabetic mice.

### Gluconeogenesis is reduced in db/db-PTG mice

**Figure 4:**
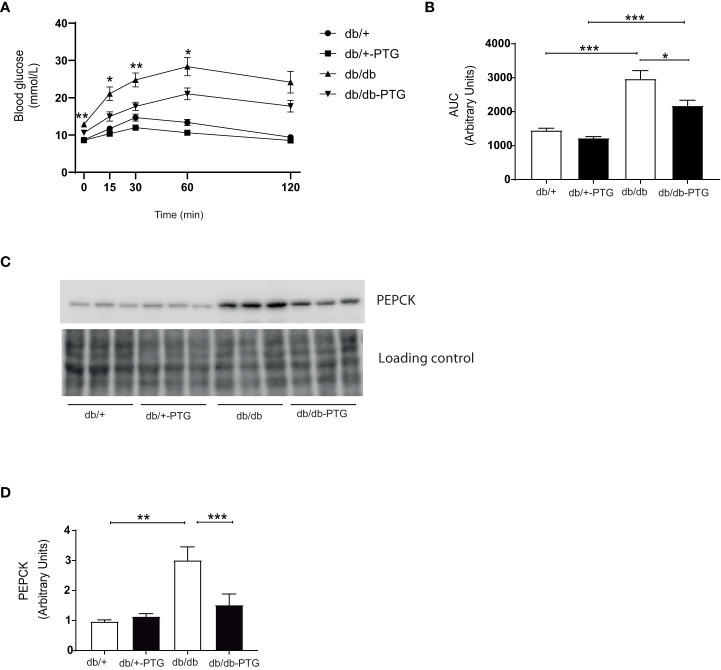
Gluconeogenesis is reduced in db/db-PTG mice. **(A)** PTT curve for glucose, **(B)** AUC for glucose in the PTT, **(C)** Representative western blot image of PEPCK and loading control, and **(D)** Quantification of PEPCK protein expression in db/db and db/db-PTG mice, and non-diabetic controls (n=5-8 in all experiments). All values are mean ± SEM. *P<0.05, **P<0.01, ***P<0.001. For figure **(A)** time 0, Diabetes factor (F ratio=49.29, P<0.0001), PTG factor (F ratio=7.396, P=0.0122), Interaction (F ratio=6.007, P=0.0223). For figure **(A)** time 15, Diabetes factor (F ratio=32.91, P<0.0001), PTG factor (F ratio=9.341, P=0.0056), Interaction (F ratio=3.774, P=0.0644). For figure **(A)** time 30, Diabetes factor (F ratio=34.99, P<0.0001), PTG factor (F ratio=13.54, P=0.0012), Interaction (F ratio=2.701, P=0.1139). For figure **(A)** time 60, Diabetes factor (F ratio=58.74, P<0.0001), PTG factor (F ratio=9.201, P=0.0059), Interaction (F ratio=1.874, P=0.1843). For figure **(A)** time 120, Diabetes factor (F ratio=39.75, P<0.0001), PTG factor (F ratio=3.667, P=0.0680), Interaction (F ratio=2.154, P=0.1557). For figure **(B)** Diabetes factor (F ratio=58.84, P<0.0001), PTG factor (F ratio=10.04, P=0.0043), Interaction (F ratio=2.989, P=0.0972). For figure **(D)** Diabetes factor (F ratio=17.23, P=0.0005), PTG factor (F ratio=5.117, P=0.0350), Interaction (F ratio=7.966, P=0.0105).

**Figure 5:**
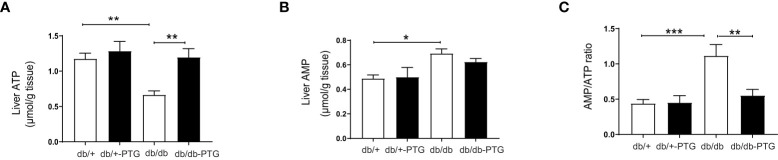
Liver PTG overexpression increases hepatic ATP levels in db/db mice. **(A)** Liver ATP content, **(B)** Liver AMP content, and **(C)** Ratio AMP : ATP ratio in db/db and db/db-PTG mice, and non-diabetic controls (n=6-8 in all experiments). All values are mean ± SEM. *P<0.05, **P<0.01, ***P<0.001. For figure **(A)** Diabetes factor (F ratio=10.26, P=0.0036), PTG factor (F ratio=9.533, P=0.0048), Interaction (F ratio=3.898, P=0.0590). For figure **(B)** Diabetes factor (F ratio=10.69, P=0.0030), PTG factor (F ratio=0.4019, P=0.5317), Interaction (F ratio=0.7271, P=0.4016). For figure **(C)** Diabetes factor (F ratio=13.08, P=0.0013), PTG factor (F ratio=6.263, P=0.0189), Interaction (F ratio=6.811, P=0.0148).

Pyruvate is a precursor of gluconeogenesis, and pyruvate tolerance test (PTT) has been used to detect subtle abnormalities in hepatic glucose production (16–18). The blood glucose concentration of mice challenged by a bolus administration of pyruvate (2 g/kg) was lower in the db/db-PTG group compared to db/db animals (**Figure 4A**). The AUC was also smaller in the formers (**Figure 4B**). We next measured the expression of phosphoenolpyruvate carboxykinase (PEPCK), one of the main gluconeogenic enzymes, by western blot. PEPCK was significantly lower in db/db-PTG compared to db/db mice (**Figures 4C, D**). PEPCK down-regulation involved a decrease in glucose output into blood, which could explain the reduction in blood glucose observed in the db/db mice overexpressing PTG. An increase in glucose production in the livers of diabetic animals has been associated with a decrease in ATP content (19). Therefore, we measured the hepatic concentration of ATP and AMP. While db/db mice had a lower ATP content compared to non-diabetic counterparts (**Figure 5A**), as previously described (19, 20), db/db-PTG mice showed similar concentrations of hepatic ATP than db/+ mice. The concentration of AMP and the AMP : ATP ratio was increased in db/db mice compared to db/+ mice (**Figures 5B, C**), but this increase was not present in the livers of db/db-PTG mice (**Figures 5B, C**).

### The expression of lipogenic genes is unaltered in db/db-PTG mice

**Figure 6:**
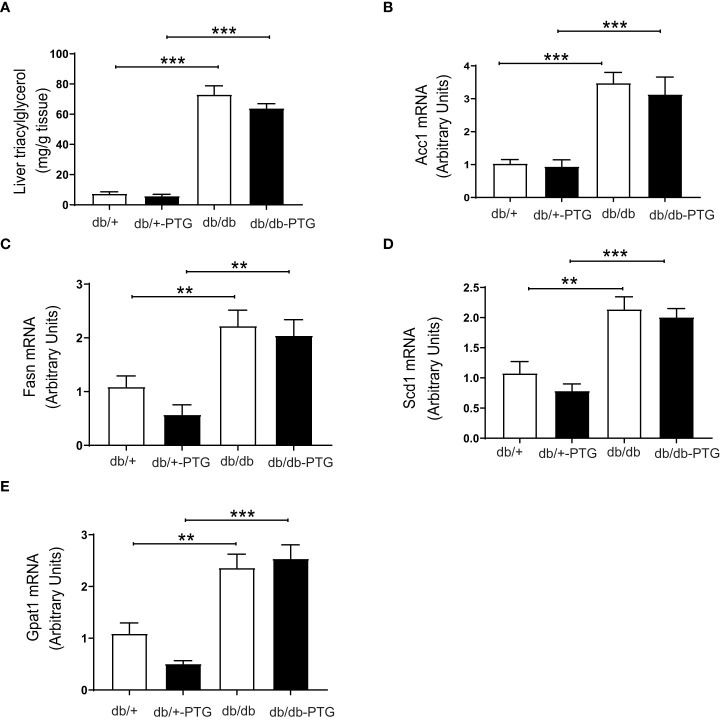
The expression of lipogenic genes is unaltered in db/db-PTG mice **(A)** Hepatic TAG content, **(B)** Hepatic ACC1 mRNA expression, **(C)** Hepatic FAS mRNA expression, **(D)** Hepatic SCD1 mRNA expression, and **(E)** Hepatic GPAT1 mRNA expression of db/db and db/db-PTG mice, and non-diabetic controls (n=6-12 in all experiments). All values are mean ± SEM. **P<0.01, ***P<0.001. For figure **(A)** Diabetes factor (F ratio=219.5, P<0.0001), PTG factor (F ratio=1.638, P=0.2095), Interaction (F ratio=0.8095, P=0.3748). For figure **(B)** Diabetes factor (F ratio=73.11, P<0.0001), PTG factor (F ratio=0.7406, P=0.3992), Interaction (F ratio=0.2758, P=0.6050). For figure **(C)** Diabetes factor (F ratio=26.50, P<0.0001), PTG factor (F ratio=1.910, P=0.1815), Interaction (F ratio=0.4528, P=0.5083). For figure **(D)** Diabetes factor (F ratio=46.03, P<0.0001), PTG factor (F ratio=1.592, P=0.2216), Interaction (F ratio=0.2301, P=0.6367). For figure **(E)** Diabetes factor (F ratio=55.54, P<0.0001), PTG factor (F ratio=0.8379, P=0.374), Interaction (F ratio=2.910, P=0.1028).

The liver is a key tissue for lipid metabolism. Consistent with genetic obesity, hepatic TAG concentration was increased in db/db mice compared to non-diabetic mice. TAG accumulation was similar in db/db and db/db-PTG animals (**Figure 6A**). We then measured the expression of genes involved in the lipogenic pathway. In this regard, the expression of acetyl-CoA carboxylase (ACC1) (**Figure 6B**), fatty acid synthase (FAS) (**Figure 6C**), stearoyl-CoA desaturase (SCD1) (**Figure 6D**) and glycerol-3-phosphate acyltransferase 1 (GPAT1) (**Figure 6E**) was higher in db/db and db/db-PTG compared to db/+ and db/+-PTG animals, respectively, and PTG overexpression did not alter the expression of these lipogenic genes in diabetic and non-diabetic mice.

## Discussion

Here we show that an increase in liver glycogen attenuated obesity in db/db mice, a model of T2DM. Similar observations have been made in an HFD mouse, another model of T2DM (8). In both cases, the decrease in body weight was accompanied by a reduction in daily food intake. An increase in energy expenditure could also protect the animals against weight gain and obesity. However, our data indicate that energy expenditure was not increased in db/db-PTG mice, thereby suggesting that the weight-reducing effect was caused exclusively by a diminished appetite. Liver ATP is a key metabolite in the regulation of food intake (21, 22). An increase in the hepatic AMP : ATP ratio stimulates food intake by vagus nerve signalling to the brain (23). In this regard, liver ATP is diminished in obesity and insulin resistance in humans (23). ATP content was significantly reduced in the livers of db/db mice, as previously described (19), but PTG overexpression restored hepatic ATP content in db/db-PTG mice. Therefore, increased liver glycogen stores, through the maintenance of hepatic ATP levels, contributed to decreasing appetite in db/db mice and this effect was probably triggered by the vagus nerve (24). A potential limitation of this study is the lack of a pair-feeding control, which would have provided information about the effect of food intake in all the parameters measured, independently of PTG overexpression. However, the present results are in line with our previous reports on the effects of the increase of hepatic glycogen in mouse models of T2DM (8, 24).

Increased liver glycogen had a marked glucose-lowering effect in db/db mice, both in fed and fasted conditions, but not in non-diabetic animals. Thus, the glucose-lowering effect of PTG overexpression was observed only in conditions of hyperglycemia, which represents an advantage from a therapeutic perspective. This glucose-lowering effect was independent of insulin since PTG overexpression did not modify plasma insulin concentration or insulin resistance.

Enhanced liver glycogenesis improves glucose homeostasis in several models of type 1 diabetes (T1DM) and T2DM (8, 14, 25). This effect is mediated not only by the diversion of blood glucose toward glycogen synthesis but also by inhibition of hepatic gluconeogenesis. In this regard, PTG overexpression in db/db mice resulted in lower expression of PEPCK, the key enzyme in the regulation of gluconeogenesis, and decreased hepatic glucose output, as determined by the flux from pyruvate to glucose *in vivo*. In line with these observations, partial silencing of hepatic PEPCK enhances glycemia in db/db mice (20).

In addition, several studies have shown that a decrease in ATP content is associated with an increase in glucose production in the livers of diabetic animals and humans (19, 25–27). In summary, PTG overexpression increased hepatic ATP levels and suppressed gluconeogenesis in db/db mice, thereby leading to a decrease in both food intake and glycemia.

Lipid accumulation in the liver is a characteristic of the diabetic phenotype in db/db mice. Caloric restriction reverts hepatic steatosis and reduces the expression of lipogenic enzymes in the livers of db/db mice (28). However, db/db-PTG mice did not show changes in the concentration of hepatic TAG or in the expression of lipogenic genes. Therefore, lipid metabolism was not improved in these mice, probably because the reduction in food intake was not as marked as in calorie-restricted db/db mice (4.8 g per day vs. 2 g per day).

In conclusion, increased liver glycogen stores improve the diabetic phenotype in db*/*db mice. Similar results were previously obtained in another model of T2DM, namely the HFD mouse (8). Furthermore, the beneficial effects of the increase in hepatic glycogen have also been demonstrated in an animal model of insulin-deficient and monogenic diabetes, namely the Akita mouse (25). On the basis of the findings of all the aforementioned studies, hepatic glycogen content emerges as a potential target for the treatment of diabetes.

## Data availability statement

The data are available on request from the corresponding author.

## Ethics statement

The animal study was reviewed and approved by Barcelona Science Park’s Animal Experimentation Committee.

## Author contributions

IL-S researched data. IL-S wrote the manuscript. JD designed the PTG overexpressing mouse. IL-S and JG designed the study. IL-S, JD, and JG revised the manuscript. IL-S is the guarantor of this work and, as such, had full access to all the data in the study and takes responsibility for the integrity of the data and the accuracy of the data analysis. The authors declare that they have no conflict of interest in relation to this work.

## Funding

This study was supported by grants from the Spanish Ministry of Science, Innovation, and Universities (MCIU/FEDER/AEI) (BFU2017-84345-P to JG and JD and PID2020-118699GB-I00 to JD), the CIBER de Diabetes y Enfermedades Metabólicas Asociadas (ISCIII, Ministerio de Ciencia e Innovación), and “La Marató de TV3” Foundation (Barcelona, Spain) (project 201613-10). We gratefully acknowledge institutional funding from the Spanish Ministry of Science and Innovation through the Centres of Excellence Severo Ochoa Award and from the CERCA Programme/Generalitat de Catalunya.

## Acknowledgments

We wish to thank Anna Adrover, Emma Veza, Vanessa Hernandez, and Laura I. Alcaide for technical assistance. We also thank Tanya Yates for correcting the English version of the manuscript.

## Conflict of interest

The authors declare that the research was conducted in the absence of any commercial or financial relationships that could be construed as a potential conflict of interest.

## Publisher’s note

All claims expressed in this article are solely those of the authors and do not necessarily represent those of their affiliated organizations, or those of the publisher, the editors and the reviewers. Any product that may be evaluated in this article, or claim that may be made by its manufacturer, is not guaranteed or endorsed by the publisher.

## Abbreviations

PTG, protein targeting to glycogen; HFD, high-fat diet; GTT, glucose tolerance test; ITT, insulin tolerance test; PTT, pyruvate tolerance test; AUC, area under the curve; PEPCK, phosphoenolpyruvate carboxykinase; T2DM, type 2 diabetes mellitus; PP1, protein phosphatase 1; Lepr, leptin receptor; RER, respiratory exchange ratio; TAG, triacylglycerol; ACC1 acetyl-CoA carboxylase 1; FAS, fatty acid synthase; SCD1, stearoyl-CoA desaturase; GPAT1, Glycerol-3-phosphate acyltransferase 1; ATPSβ, ATP synthase β.

## References

[1] Unnikrishnan R Pradeepa R Joshi SR Mohan V. Type 2 diabetes: Demystifying the global epidemic. Diabetes (2017) 66(6):1432–42. doi: 10.2337/db16-0766 28533294

[2] Khan MAB Hashim MJ King JK Govender RD Mustafa H Al Kaabi J. Epidemiology of type 2 diabetes - global burden of disease and forecasted trends. J Epidemiol Global Health (2020) 10(1):107–11. doi: 10.2991/jegh.k.191028.001 PMC7310804 32175717

[3] Han HS Kang G Kim JS Choi BH Koo SH. Regulation of glucose metabolism from a liver-centric perspective. Exp Mol Med (2016) 48:e218. doi: 10.1038/emm.2015.122 26964834 PMC4892876

[4] Petersen MC Vatner DF Shulman GI. Regulation of hepatic glucose metabolism in health and disease. Nat Rev Endocrinol (2017) 13(10):572–87. doi: 10.1038/nrendo.2017.80 PMC5777172 28731034

[5] Krssak M Brehm A Bernroider E Anderwald C Nowotny P Dalla Man C. Alterations in postprandial hepatic glycogen metabolism in type 2 diabetes. Diabetes (2004) 53(12):3048–56. doi: 10.2337/diabetes.53.12.3048 15561933

[6] Printen JA Brady MJ Saltiel AR. PTG, a protein phosphatase 1-binding protein with a role in glycogen metabolism. Science (1997) 275(5305):1475–8. doi: 10.1126/science.275.5305.1475 9045612

[7] O'Doherty RM Jensen PB Anderson P Jones JG Berman HK Kearney D. Activation of direct and indirect pathways of glycogen synthesis by hepatic overexpression of protein targeting to glycogen. J Clin Invest (2000) 105(4):479–88. doi: 10.1172/JCI8673 PMC289167 10683377

[8] Lopez-Soldado I Zafra D Duran J Adrover A Calbo J Guinovart JJ. Liver glycogen reduces food intake and attenuates obesity in a high-fat diet-fed mouse model. Diabetes (2015) 64(3):796–807. doi: 10.2337/db14-0728 25277398

[9] Lee GH Proenca R Montez JM Carroll KM Darvishzadeh JG Lee JI. Abnormal splicing of the leptin receptor in diabetic mice. Nature (1996) 379(6566):632–5. doi: 10.1038/379632a0 8628397

[10] Parekh PI Petro AE Tiller JM Feinglos MN Surwit RS. Reversal of diet-induced obesity and diabetes in C57BL/6J mice. Metabolism: Clin experimental (1998) 47(9):1089–96. doi: 10.1016/s0026-0495(98)90283-9 9751238

[11] Lopez-Soldado I Guinovart JJ Duran J. Increased liver glycogen levels enhance exercise capacity in mice. J Biol Chem (2021) 297(2):100976. doi: 10.1016/j.jbc.2021.100976 34284060 PMC8350413

[12] Salmon DM Flatt JP. Effect of dietary fat content on the incidence of obesity among ad libitum fed mice. Int J Obes (1985) 9(6):443–9. 3830936

[13] Roesler WJ Khandelwal RL. Age-related changes in hepatic glycogen metabolism in the genetically diabetic (db/db) mouse. Diabetes (1985) 34(4):395–402. doi: 10.2337/diab.34.4.395 2982686

[14] Ros S Zafra D Valles-Ortega J Garcia-Rocha M Forrow S Dominguez J. Hepatic overexpression of a constitutively active form of liver glycogen synthase improves glucose homeostasis. J Biol Chem (2010) 285(48):37170–7. doi: 10.1074/jbc.M110.157396 PMC2988323 20841354

[15] Kobayashi K Forte TM Taniguchi S Ishida BY Oka K Chan L. The db/db mouse, a model for diabetic dyslipidemia: molecular characterization and effects of Western diet feeding. Metabolism: Clin experimental (2000) 49(1):22–31. doi: 10.1016/S0026-0495(00)90588-2 10647060

[16] Rodgers JT Puigserver P. Fasting-dependent glucose and lipid metabolic response through hepatic sirtuin 1. Proc Natl Acad Sci U S A (2007) 104(31):12861–6. doi: 10.1073/pnas.0702509104 PMC1937557 17646659

[17] Miyake K Ogawa W Matsumoto M Nakamura T Sakaue H Kasuga M. Hyperinsulinemia, glucose intolerance, and dyslipidemia induced by acute inhibition of phosphoinositide 3-kinase signaling in the liver. J Clin Invest (2002) 110(10):1483–91. doi: 10.1172/JCI0215880 PMC151813 12438446

[18] Lopez-Soldado I Bertini A Adrover A Duran J Guinovart JJ. Maintenance of liver glycogen during long-term fasting preserves energy state in mice. FEBS Lett (2020) 594(11):1698–710. doi: 10.1002/1873-3468.13770 PMC7282949 32159852

[19] Wang C Chen Z Li S Zhang Y Jia S Li J. Hepatic overexpression of ATP synthase beta subunit activates PI3K/Akt pathway to ameliorate hyperglycemia of diabetic mice. Diabetes (2014) 63(3):947–59. doi: 10.2337/db13-1096 24296716

[20] Gomez-Valades AG Mendez-Lucas A Vidal-Alabro A Blasco FX Chillon M Bartrons R. Pck1 gene silencing in the liver improves glycemia control, insulin sensitivity, and dyslipidemia in db/db mice. Diabetes (2008) 57(8):2199–210. doi: 10.2337/db07-1087 PMC2494684 18443203

[21] Ritter S Dinh TT Friedman MI. Induction of fos-like immunoreactivity (Fos-li) and stimulation of feeding by 2,5-anhydro-D-mannitol (2,5-AM) require the vagus nerve. Brain Res (1994) 646(1):53–64. doi: 10.1016/0006-8993(94)90057-4 8055341

[22] Grill HJ Friedman MI Norgren R Scalera G Seeley R. Parabrachial nucleus lesions impair feeding response elicited by 2,5-anhydro-D-mannitol. Am J Physiol (1995) 268(3 Pt 2):R676–82. doi: 10.1152/ajpregu.1995.268.3.R676 7900910

[23] Cooper ME Regnell SE. The hepatic cannabinoid 1 receptor as a modulator of hepatic energy state and food intake. Br J Clin Pharmacol (2014) 77(1):21–30. doi: 10.1111/bcp.12102 23452341 PMC3895344

[24] Lopez-Soldado I Fuentes-Romero R Duran J Guinovart JJ. Effects of hepatic glycogen on food intake and glucose homeostasis are mediated by the vagus nerve in mice. Diabetologia (2017) 60(6):1076–83. doi: 10.1007/s00125-017-4240-4 28299379

[25] Lopez-Soldado I Guinovart JJ Duran J. Increasing hepatic glycogen moderates the diabetic phenotype in insulin-deficient akita mice. J Biol Chem (2021) 296:100498. doi: 10.1016/j.jbc.2021.100498 33667544 PMC8027280

[26] Schmid AI Szendroedi J Chmelik M Krssak M Moser E Roden M. Liver ATP synthesis is lower and relates to insulin sensitivity in patients with type 2 diabetes. Diabetes Care (2011) 34(2):448–53. doi: 10.2337/dc10-1076 PMC3024365 21216854

[27] Szendroedi J Chmelik M Schmid AI Nowotny P Brehm A Krssak M. Abnormal hepatic energy homeostasis in type 2 diabetes. Hepatology (2009) 50(4):1079–86. doi: 10.1002/hep.23093 19637187

[28] Kim KE Jung Y Min S Nam M Heo RW Jeon BT. Caloric restriction of db/db mice reverts hepatic steatosis and body weight with divergent hepatic metabolism. Sci Rep (2016) 6:30111. doi: 10.1038/srep30111 27439777 PMC4954985

